# Factors influencing quality of life in extratemporal lobe epilepsy and mesial temporal lobe epilepsy: a cross-sectional study using medical records

**DOI:** 10.3389/fneur.2024.1443903

**Published:** 2024-07-29

**Authors:** Hiroki Annaka, Tomonori Nomura, Naoya Hasegawa

**Affiliations:** ^1^Department of Occupational Therapy, Faculty of Rehabilitation, Niigata University of Health and Welfare, Niigata, Japan; ^2^Department of Psychiatry, National Hospital Organization, Nishiniigata Chuo Hospital Epilepsy Center, Niigata, Japan

**Keywords:** quality of life, epilepsy, social participation, seizure, antiseizure medication

## Abstract

**Objective:**

This study aimed to examine differences in factors influencing quality of life (QOL) in people with extratemporal lobe epilepsy (ETLE) and mesial temporal lobe epilepsy (MTLE).

**Methods:**

We obtained data from the medical records of 84 (47 ETLE and 37 MTLE) people with epilepsy. The data included age, sex, employment, seizure frequency, number of antiseizure medication (ASM), Neurological Disorders Depression Inventory for Epilepsy (NDDI-E) score, and Quality of Life in Epilepsy Inventory-31 (QOLIE-31) score. Multiple regression analyses were performed using QOLIE-31 as the dependent variable and age, sex, employment, seizure frequency, number of ASM, and NDDI-E score as the independent variables in ETLE or MTLE.

**Results:**

From the results of the multiple regression analyses, QOLIE-31 in ETLE was associated with NDDI-E (*β* = −0.757, *p* < 0.001) and employment (*β* = 0.388, *p* = 0.008). Meanwhile, QOLIE-31 in MTLE was associated with NDDI-E (*β* = −0.625, *p* < 0.001), employment (*β* = 0.396, *p* = 0.041), and number of ASMs (*β* = −0.399, *p* = 0.018).

**Conclusion:**

Overall, our findings indicate that the number of ASMs is potentially an influence on QOL of MTLE but similar effect is not observed in ETLE.

## 1 Introduction

Epilepsy has neurological, psychological, and social consequences that affect the quality of life (QOL) of people with epilepsy (PWE) (1). QOL in PWE is extremely low compared to that in other chronic diseases (2). QOL can be broadly defined as a multidimensional concept that describes the relationship between physical, psychological, social, and environmental factors (1, 3). Identification of factors influencing QOL in PWE is essential to support the development of interventions that can improve overall QOL.

Previous studies have explored the factors influencing the QOL in PWE (1). Depression is comorbid in 22.9% of PWE and is a strong influencing factor in QOL (4). Poor seizure control, including the frequency of seizures and antiseizure medication (ASM) polypharmacy, affects QOL (4, 5). The majority of PWE are in a situation of unemployment (6). Employment is an important factor to promote psychosocial adjustment in PWE (5). However, previous studies have not fully explored the factors influencing QOL based on the type of focal epilepsy, such as extratemporal lobe epilepsy (ETLE) or mesial temporal lobe epilepsy (MTLE).

The MTLE is a focal seizure in the limbic system, such as the hippocampus and amygdala, which causes uncontrolled seizures with impaired awareness, resulting in impaired mental and physical health (7, 8). Conversely, ETLE is a focal seizure in the cerebral cortex, excluding the hippocampus and amygdala, such as the frontal, parietal, and lateral temporal lobes, and has different symptoms depending on the focal point. Previous studies have compared social cognition and psychiatric comorbidity between ETLE and MTLE (9, 10); however, only few have examined differences in factors influencing QOL. This study aimed to examine differences in factors influencing QOL in people with ETLE and MTLE. We hypothesized that these factors differ between ETLE and MTLE.

## 2 Methods

### 2.1 Design

We used a cross-sectional design for this study, which was based on the REporting of studies conducted using the Observational Routinely-collected health Data (RECORD) Statement (11). Data were collected from medical records of PWE in the outpatient clinic at the Department of epilepsy medicine, National Hospital Organization Nishiniigata Chuo Hospital, from June 1, 2021, to December 31, 2021. The medical records comprised patients’ medical history and social and medical information. Collected data included age, sex, education, marital status, employment, duration of epilepsy, seizures (duration, frequency, and type), ASM used, Neurological Disorders Depression Inventory for Epilepsy (NDDI-E) score, and Quality of Life in Epilepsy Inventory-31 (QOLIE-31) score. Patients completed the self-administered NDDI-E and QOLIE-31 while waiting for their consultation, and the occupational therapist (H.A.) interpreted and entered the results into medical records. A physician (N.H.) collected other data, except for NDDI-E and QOLIE-31 scores, and recorded them in medical records.

### 2.2 Medical records data

This study included individuals with a diagnosis of epilepsy (based on the 10th revision of the International Statistical Classification of Diseases code: G40). The inclusion criteria were as follows: (1) age of 18–60 years and (2) diagnosis of ETLE or MTLE. ETLE or MTLE was diagnosed by a physician (N.H). Magnetic resonance imaging (MRI) findings, electroencephalogram (EEG) findings, and seizure semiology were classified as MTLE origin or other (frontal lobe, lateral temporal lobe, parietal lobe, and occipital lobe) origin according to the diagnostic manual of The International League Against Epilepsy Position paper regarding MTLE or ETLE diagnosis (12). Only patients whose MRI, EEG, and seizure semiology findings two or more were diagnosed with MTLE or ETLE. Supplementary Table S1 shows the seizure semiology in MTLE. The exclusion criteria were as follows: (1) undergoing epilepsy surgery, (2) diagnosis of a mental disorder including depression, developmental disorder, intellectual disability, and other neurological disorders, (3) taking psychiatric medication, (4) inability to read Japanese, and (5) patients with missing data. Additionally, patients with both MTLE and ETLE findings were considered to have focal epilepsy with dual pathology and were excluded from this study.

### 2.3 Ethics statement

The ethics committee of the National Hospital Organization Nishiniigata Chuo Hospital approved the study protocol and use of medical record data (approval no. 2211). The Ethics Committee determined that informed consent was not required because this study would use only anonymized data from medical records. All medical records data were completely anonymized and de-identified. We accessed the medical records on March 23, 2022, and did not access any personally identifiable information during and after data collection. The Declaration of Helsinki was observed in the conduct of the study.

### 2.4 Measurement

We used the QOLIE-31 to measure the QOL of PWE (13). The QOLIE-31 comprises 31 items across seven subscales: seizure worry, overall QOL, emotional well-being, energy/fatigue, cognitive functioning, medication effects, and social functioning. All items were used to calculate the total score. Total and subscale scores were calculated with reference to the QOLIE-31 scoring manual (14). Each score ranges from 0 to 100, with higher scores indicating better QOL.

We used the NDDI-E to measure depression symptoms in PWE (15). This questionnaire consists of 6 items and is scored in a range of 6–24 points. A higher score indicates more severe depression symptoms.

### 2.5 Statistical analysis

Statistical analysis was conducted using IBM Statistical Package for the Social Sciences Statistics for Windows, Version 20.0 (IBM Japan, Tokyo, Japan). The mean and standard deviation (SD) or median and interquartile range (IQR) or number and percentage (%) of all variables are presented. The normal distribution of each continuous variable was confirmed using the Shapiro–Wilk test. The Mann–Whitney *U* test or chi-square test was used to compare all variables and QOLIE-31 (total score and each domain score) between a group of ETLE or MTLE. Multiple regression analysis (least-squares method) was used to identify factors associated with the total QOLIE-31 score (dependent variable) in the ETLE or MTLE groups. The normality of the residuals of the total QOLIE-31 score in the ETLE or MTLE groups was confirmed before the analysis using a quantile–quantile plot (QQ plot). The algorithm for variable selection was dependent on background knowledge, and variable selection was performed through forced entry. Independent variables in this study, including employment (0: no, 1: yes) (5), seizure frequency (0: non-seizure, 1: per year, 2: per month, 3: per day) (5), number of ASMs (continuous variable) (4), and NDDI-E (continuous variable) (4, 5), were determined by referring to a previous study, and covariates included age and sex. In the analysis, independent variables were excluded if neither of the two conditions was applicable. Condition one is that the correlation with QOLIE-31 has a *p*-value of <0.05 in the Spearman rank correlation coefficient. Condition two is a variable wherein the median QOLIE-31 total score for each group is divided into high and low groups, and comparisons from the Mann–Whitney *U* test or chi-square test have a *p*-value <0.05. Age and sex were included as covariates as these influencing factors are associated with epilepsy, including QOL (16–18). One independent variable and covariates (age and sex) were used for the analysis. In addition, when the extracted factors differed between ETLE and MTLE, we examined the causes through multiple regression analysis, following the above-mentioned algorithm, with each domain of the QOLIE-31 as a dependent variable. Multicollinearity was considered to be present if the variance inflation factor was ≥10. The level of significance was set at a *p*-value of <0.05.

### 2.6 Sample size

The multiple regression analysis was conducted to determine the sample size. The sample size was calculated using G*Power version 3.1.9.4 (Heinrich-Heine-Universität Düsseldorf, Düsseldorf, Germany). The test family included *F*-tests, with a statistical test of linear multiple regression with a fixed model, *R*^2^ deviation from zero, an effect size of *f*^2^ 0.35, *α* err prob. of 0.05, power of 0.8, and predictors of 3 (19). The effect size was calculated using the following formula, referring to a previous study (9):


$$f^{2}=\frac{R^{2}}{1-R^{2}}$$

The required sample size for this study was determined to be >36 individuals.

## 3 Results

### 3.1 The characteristics of subjects

This study included 84 PWE, including 47 with ETLE and 37 with MTLE (Table 1). All continuous variables were non-normally distributed. The median age was 41.0 (32.0–48.0) years, with 39 men (46%) and 45 women (54%). The median seizure onset was 15.0 (11.0–21.0) years, and the median seizure duration was 15.5 (5.0–25.0) years. Controlled seizures were observed in 54 (64%) patients. A comparison between the ETLE and MTLE groups showed no difference in age (*p* = 0.283), sex (*p* = 0.510), employment (*p* = 0.279), seizure frequency (none: *p* = 0.820, per year: *p* = 1.000, per month: *p* = 0.115, per day: *p* = 0.127), number of ASMs used (*p* = 0.379), and NDDI-E score (*p* = 0.622). Perampanel administration was higher in the ETLE group than in the MTLE group (*p* = 0.015) (Table 2).

**Table 1:** Patient characteristics.

Characteristics	Overall (*n* = 84)	Extratemporal lobe epilepsy (*n* = 47)	Mesial temporal lobe epilepsy (*n* = 37)	*p*-value
Age (years)	41.0 (32.0–48.0)	37.00 (27.0–46.0)	44.0 (37.0–49.0)	0.283
Sex				
Male	39 (46%)	20 (42%)	19 (51%)	0.510
Female	45 (54%)	27 (58%)	18 (49%)	0.510
Education (years)	13.0 (12.0–14.0)	12.00 (12.0–14.0)	14.0 (12.0–16.0)	0.560
Marital status	32 (38%)	15 (31%)	17 (45%)	0.258
Employment	68 (80%)	36 (76%)	32 (86%)	0.279
Seizure onset (year)	15.0 (11.0–21.0)	15.0 (11.0–20.0)	19.0 (9.0–26.0)	0.527
Duration of epilepsy (year)	22.0 (12.2–32.7)	23.0 (11.0–33.0)	22.0 (12.5–33.0)	1.000
Duration of seizures (year)	15.5 (5.0–25.0)	17.0 (5.0–24.0)	14.0 (3.0–27.5)	0.806
Seizure frequency				
None	54 (64%)	31 (65%)	23 (62%)	0.820
Per year	14 (14%)	3 (6%)	9 (24%)	1.000
Per month	15 (17%)	10 (21%)	5 (13%)	0.115
Per day	4 (4%)	4 (8%)	0 (0%)	0.127
Type of seizure				
Focal aware	10 (11%)	6 (12%)	4 (10%)	1.000
FIAS	22 (26%)	11 (23%)	11 (29%)	0.619
Generalized	52 (61%)	30 (63%)	22 (59%)	0.462
Number of ASMs	1.5 (1.0–3.0)	2.0 (1.0–3.0)	1.0 (1.0–2.0)	0.379
Polypharmacy	42 (50%)	26 (55%)	16 (43%)	0.379
NDDI-E score	11.0 (8.0–14.7)	12.0 (9.0–15.0)	11.0 (7.5–14.5)	0.622

FIAS, focal impaired awareness; ASM, antiseizure medication; NDDI-E, neurological disorders depression inventory for epilepsy. Median (interquartile range) or number (%). Mann–Whitney *U* test or chi-square test. *p* < 0.05.

**Table 2:** Antiseizure medication profile.

Antiseizure medications	Overall (*n* = 84)	Extratemporal lobe epilepsy (*n* = 47)	Mesial temporal lobe epilepsy (*n* = 37)	*p*-value
Lacosamide	9 (10%)	4 (8%)	5 (13%)	0.498
Gabapentin	2 (2%)	1 (2%)	1 (2%)	1.000
Phenobarbital	2 (2%)	1 (2%)	1 (2%)	1.000
Clonazepam	1 (1%)	0 (0%)	1 (2%)	0.440
Phenytoin	15 (17%)	8 (17%)	7 (18%)	1.000
Carbamazepine	20 (23%)	8 (17%)	12 (32%)	0.125
Topiramate	3 (3%)	2 (4%)	1 (2%)	1.000
Sodium valproate	14 (16%)	11 (23%)	3 (8%)	0.080
Levetiracetam	40 (47%)	24 (51%)	16 (43%)	0.515
Zonisamide	4 (4%)	2 (4%)	2 (5%)	1.000
Clobazam	10 (11%)	7 (8%)	3 (8%)	0.501
Clonazepam	1 (1%)	0 (0%)	1 (2%)	0.440
Lamotrigine	18 (21%)	10 (21%)	8 (21%)	1.000
Perampanel	17 (20%)	14 (29%)	3 (8%)	0.015

Number (%). Chi-square test. *p* < 0.05.

### 3.2 Comparison of the QOLIE-31 scores

The QOLIE-31 scores were as follows: the total score was 70.6 (13.9), seizure worry score was 61.7 (23.5), emotional well-being score was 64.9 (18.0), overall QOL score was 55.8 (15.0), energy/fatigue score was 63.1 (15.6), cognitive functioning score was 83.6 (18.0), medical effect score was 60.1 (10.6), and social functioning score was 77.5 (26.2). The QOLIE-31 total score and each domain scores did not differ between the groups (Table 3); total score (*p* = 0.079), seizure worry (*p* = 0.846), emotional well-being (*p* = 0.698), overall QOL (*p* = 0.466), energy/fatigue (*p* = 0.527), cognitive functioning (*p* = 0.660), medical effects (*p* = 0.187), and social functioning (*p* = 0.187).

**Table 3:** Comparison of the QOLIE-31 score between the extratemporal lobe epilepsy and mesial temporal lobe epilepsy groups.

QOLIE-31	Overall (*n* = 84)	Extratemporal lobe epilepsy (*n* = 47)	Mesial temporal lobe epilepsy (*n* = 37)	*p*-value
Total score	70.6 (13.9)	68.1 (14.9)	73.1 (12.1)	0.079
Seizure worry	61.7 (23.5)	59.7 (24.6)	64.2 (22.1)	0.846
Emotional well-being	64.9 (18.0)	64.9 (18.4)	66.5 (17.8)	0.698
Overall QOL	55.8 (15.0)	54.6 (14.4)	57.2 (15.8)	0.466
Energy/fatigue	63.1 (15.6)	62.5 (15.1)	63.9 (16.3)	0.527
Cognitive functioning	83.6 (18.0)	81.5 (19.5)	86.2 (15.8)	0.660
Medical effects	60.1 (10.6)	60.1 (9.8)	60.2 (11.7)	0.187
Social functioning	77.5 (26.2)	73.4 (27.5)	82.7 (23.6)	0.187

QOLIE-31, quality of life in epilepsy inventory-31. Mean (standard deviation). Mann–Whitney *U* test. *p* < 0.05.

### 3.3 Multiple regression analysis of the total QOLIE-31 score in ETLE

The residuals of the total score of QOLIE-31 in ETLE showed normality. The variables were employment, ASM, and NDDI-E, selected by Spearman rank correlation coefficient (Supplementary Table S2) and Mann–Whitney *U* test or chi-square test (Supplementary Table S3). The total score obtained in multiple regression analysis according to the ETLE group was associated with employment (*β* = 0.388, *R*^2^ = 0.205, *p* = 0.008) and NDDI-E score (*β* = −0.757, *R*^2^ = 0.598, *p* < 0.001) (Table 4).

**Table 4:** Multiple regression analysis according to the extratemporal lobe epilepsy group (*n* = 47).

Variable	B	SE	β	t	*R* ^2^	*p*-value	95% CI		VIF
							Lower	Upper	
Total QOLIE-31 score									
Employment	13.051	4.664	0.388	2.798	0.205	0.008	3.645	22.457	1.040
Number of ASMs	−3.274	0.191	−0.246	−1.622	0.115	0.112	−7.343	0.796	1.086
NDDI-E score	−2.690	0.355	−0.757	−7.580	0.598	<0.001	−3.405	−1.974	1.066

Dependent variable: total Quality of Life in Epilepsy Inventory-31 score. Independent variable: employment (0: no; 1: yes); number of ASMs (continuous variable); NDDI-E score (continuous variable). Covariates: age (continuous variable); sex (1: male; 2: female). Each independent variable was adjusted for age and sex. B, partial regression coefficient; SE, standard error; β, standardized partial regression coefficient; *R*^2^, coefficient of determination; 95% CI, 95% confidence interval; VIF, variance inflation factor; NDDI-E, Neurological Disorders Depression Inventory for Epilepsy.

### 3.4 Multiple regression analysis of the total QOLIE-31 score in MTLE

The residuals of the total QOLIE-31 score in MTLE showed normality. The variables were employment, ASM, and NDDI-E, selected by Spearman rank correlation coefficient (Supplementary Table S4) and Mann–Whitney *U* test or chi-square test (Supplementary Table S5). The total score obtained in multiple regression analysis according to the MTLE group was associated with the employment (*β* = 0.396, *R*^2^ = 0.122, *p* = 0.041), number of ASMs used (*β* = −0.399, *R*^2^ = 0.160, *p* = 0.018), and NDDI-E score (*β* = −0.625, *R*^2^ = 0.367, *p* < 0.001) (Table 5).

**Table 5:** Multiple regression analysis according to the mesial temporal lobe epilepsy group (*n* = 37).

Variable	*B*	*SE*	*β*	*t*	*R* ^2^	*p*-value	95% CI		VIF
							Lower	Upper	
Total QOLIE-31 score									
Employment	15.042	7.705	0.396	2.126	0.122	0.041	0.649	29.436	1.302
Number of ASMs	−5.605	2.246	−0.399	−2.495	0.160	0.018	−10.174	−1.035	1.007
NDDI-E score	−1.906	0.437	−0.625	−4.360	0.367	<0.001	−2.796	−1.017	1.070

Dependent variable: total Quality of Life in Epilepsy Inventory-31 score. Independent variable: employment (0: no; 1: yes); number of ASMs (continuous variable); NDDI-E score (continuous variable). Covariates: age and sex (1: male; 2: female). Each independent variable was adjusted for age and sex. B, partial regression coefficient; SE, standard error; β, standardized partial regression coefficient; *R*^2^, coefficient of determination; 95% CI, 95% confidence interval; VIF, variance inflation factor; ASM, antiseizure medication; NDDI-E, Neurological Disorders Depression Inventory for Epilepsy.

### 3.5 Association between the number of ASMs and each domain of QOLIE-31 scores

The number of ASMs was identified as a factor influencing QOL only for MTLE. Multiple regression analysis was performed with each domain of the QOLIE-31 as an independent variable and the number of ASMs as a dependent variable in the ETLE or MTLE groups.

Independent variables in the ETLE group were selected using the Spearman rank correlation coefficient (Supplementary Table S6), Mann–Whitney *U* test, or chi-squared test (Supplementary Table S7) for seizure worry and social functioning. The number of ASMs showed an association with seizure worry (*β* = −0.322, *R*^2^ = 0.173, *p* = 0.033) and social functioning (*β* = −0.292, *R*^2^ = 0.186, *p* = 0.044) in multiple regression analysis according to the ETLE group (Table 6).

**Table 6:** Multiple regression analysis using each domain of the Quality of Life in Epilepsy Inventory-31 and antiseizure medications according to the extratemporal lobe epilepsy group (*n* = 47).

Variable	*B*	*SE*	*β*	*t*	*R* ^2^	*p*-value	95% CI		VIF
							Lower	Upper	
Seizure worry									
Number of ASMs	−7.345	3.343	−0.322	−2.198	0.173	0.033	−14.086	−0.605	1.118
Social functioning									
Number of ASMs	−7.455	3.585	−0.292	−2.079	0.186	0.044	−14.686	−2.225	1.118

Dependent variable: seizure worry domain score or social functioning score. Independent variable: number of ASM (continuous variable). Covariates: age and sex (1: male; 2: female). Each independent variable was adjusted for age and sex. B, partial regression coefficient; SE, standard error; β, standardized partial regression coefficient; *R*^2^, coefficient of determination; 95% CI, 95% confidence interval; VIF, variance inflation factor; ASM, antiseizure medication.

Independent variables in the MTLE group were selected by Spearman rank correlation coefficient (Supplementary Table S8), Mann–Whitney *U* test, or chi-squared test (Supplementary Table S9) for seizure worry, emotional well-being, and social functioning. The number of ASMs showed an association with seizure worry (*β* = −0.425, *R*^2^ = 0.204, *p* = 0.010) and emotional well-being (*β* = −0.485, *R*^2^ = 0.246, *p* = 0.003) in multiple regression analysis according to the MTLE group (Table 7).

**Table 7:** Multiple regression analysis using each domain of the Quality of Life in Epilepsy Inventory-31 and antiseizure medications according to the mesial temporal lobe epilepsy group (*n* = 37).

Variable	*B*	*SE*	*β*	*t*	*R* ^2^	*p*-value	95% CI		VIF
							Lower	Upper	
Seizure worry									
Number of ASMs	−10.034	3.680	−0.425	−2.726	0.204	0.010	−17.552	−2.546	1.007
Emotional well-being									
Number of ASMs	−9.196	2.875	−0.485	−3.198	0.246	0.003	−15.046	−3.346	1.007
Social functioning									
Number of ASMs	−5.028	4.282	−0.199	−1.174	0.056	0.249	−13.739	3.683	1.007

Dependent variable: seizure worry domain score or emotional well-being score or social functioning score. Independent variable: number of ASM (continuous variable). Covariates: age and sex (1: male; 2: female). Each independent variable was adjusted for age and sex. B, partial regression coefficient; SE, standard error; β, standardized partial regression coefficient; *R*^2^, coefficient of determination; 95% CI, 95% confidence interval; VIF, variance inflation factor; ASM, antiseizure medication.

## 4 Discussion

This study examined factors influencing QOL in ETLE and MTLE. QOL scores measured using the QOLIE-31 were not different between ETLE and MTLE. QOL in ETLE was associated with depression symptoms and employment, whereas that in MTLE was associated with depression symptoms, employment, and number of ASM. These results show that the number of ASMs may influence QOL only in MTLE.

Few studies have separately investigated factors influencing QOL in patients with ETLE and MTLE. A previous study revealed that ETLE and MTLE have different psychiatric comorbidities influencing QOL, despite having equal QOL scores as measured using the MOS 36-item Short-Form Health Survey (SF-36) (9). The present study investigated factors influencing QOL in PWE, such as employment and ASM use, and identified different the factors influencing QOL between ETLE and MTLE. QOL in MTLE was influenced the number of ASMs. This factor have been reported to influencing QOL in PWE (1). In particular, refractory MTLE is associated with low QOL due to polypharmacy (20). While, the number of ASMs are unassociated with QOL in ETLE. Previous studies have not revealed association between QOL and ASM in ETLE (9), which is similar with the results of this study. In both MTLE and ETLE, the domain of seizure worry was associated with the number of ASMs. When PWE are worried about seizures, ASMs will be less likely reduced. ETLE was associated with the domain of social functioning and number of ASMs, but may have been insufficient to affect QOL. This presumably did not influence QOL due to side effects such as drowsiness and swollen gums. One possible reason for the influence of ASM only on QOL in MTLE may be the psychiatric and behavioral side effects associated with polypharmacy affecting QOL. The study results demonstrated the number of ASMs-influenced domains of emotional well-being in the MTLE group, suggesting that polypharmacy may increase the risk of depression symptoms (21). A meta-analysis reported a higher prevalence of psychiatric symptoms in temporal lobe epilepsy compared to other focal epilepsy (22). This is because the temporal lobe area may be a key brain region in psychiatric symptoms (22). Recent studies have discussed the risk of the onset of psychiatric and behavioral side effects with polypharmacy in temporal lobe epilepsy, but this has not been fully elucidated (23). We propose that the QOL, especially psychiatric and behavioral aspects, in MTLE may be more affected by the side effects including psychiatric symptoms of polypharmacy than in ETLE. However, the study did not collect data on ASM side effects. Moreover, we were also unable to examine the influence of ASM interactions in polypharmacy. Further studies are needed to discuss these confounding factors.

This study indicated that employment and depression symptoms influence QOL in both ETLE and MTLE. Many PWE are unemployed and have reduced QOL due to poverty (5). Psychosocial factors, such as individual and environmental factors, influence the association between employment and QOL in PWE (24). For instance, driving is restricted by the frequency and severity of seizures, and work performance is influenced by depression symptoms (25). These factors create a negative impression to employers and deprive PWE of employment opportunities. Depression symptoms is the strongest factor influencing QOL in PWE (4, 26). The International League Against Epilepsy Psychology Task Force recommends screening for PWE because of the risk of comorbid depression (26). The negative impact of depressive symptoms on QOL, combined with anxiety and stigma, can be significant (18, 25). Unemployment and depressive symptoms are interrelated through several socio-psychological factors. As such, these are issues that many PWE may face, influencing their QOL regardless of the type of focal epilepsy. However, it is important to note that this single-center study based on medical records has limitations such as a small sample size and the absence of certain factors such as stigma or anxiety. Therefore, multiple regression analysis may not adequately investigate the effects of confounding factors. A larger study with additional data not collected in this study could better clarify the differences in factors influencing QOL between ETLE and MTLE.

This study has several strengths. Both the ETLE and MTLE groups were matched for social and medical information. Additionally, we excluded individuals receiving surgical treatment for epilepsy. QOL data for non-surgical PWE, especially those with ETLE, have not been widely reported. Notably, 64% of PWE experienced no seizures in this study. The results regarding seizure frequency should be interpreted with caution. Meanwhile, data on QOL for PWE with controlled seizures are also scarce. Investigating the factors influencing QOL in such groups may allow for a more detailed elucidation of the influence of socio-psychological aspects.

This study also has some limitations, as discussed above. First, this was a single-center study with a small sample size. Hence, a multicentre study is warranted. Second, this study used data from the medical records; thus, data on symptoms at each focal point, psychological and behavioral, and ASM side effects were unavailable. Third, there is the possibility of selection bias due to the use of medical records data. Fourth, this study identified limited factors for cross-sectional validation; hence, causal relationships could not be determined. Fifth, we excluded patients with a diagnosis of depression and those who were taking psychiatric medications. Thus, our findings may not reflect the influence of major depression on QOL. Finally, patients with missing data were excluded from this study, which may have introduced exclusion bias. Further prospective longitudinal studies are needed to address these limitations.

## 5 Conclusion

This study examined the factors influencing QOL in people with ETLE and MTLE. The number of ASMs is potentially an influence on QOL of MTLE but similar effect is not observed in ETLE. Large multicentre study is needed to ensure generalizability of these results.

## Data availability statement

The original contributions presented in the study are included in the article/Supplementary material, further inquiries can be directed to the corresponding author.

## Ethics statement

The studies involving humans were approved by the Ethics Committee of the National Hospital Organization Nishiniigata Chuo Hospital (Approval number: 2211). The studies were conducted in accordance with the local legislation and institutional requirements. The ethics committee/institutional review board waived the requirement of written informed consent for participation from the participants or the participants' legal guardians/next of kin because this study would only use anonymized data from medical records. All medical records data were completely anonymized and de-identified.

## Author contributions

HA: Writing – review & editing, Writing – original draft, Validation, Project administration, Methodology, Investigation, Formal analysis, Conceptualization. TN: Writing – review & editing, Supervision, Methodology, Conceptualization. NH: Writing – review & editing, Project administration, Methodology, Investigation, Conceptualization.
