# Supplementary material for: Factors influencing quality of life in extratemporal lobe epilepsy and mesial temporal lobe epilepsy: a cross-sectional study using medical records
Source: Front Neurol. 2024 Jul 29;15:1443903. doi: 10.3389/fneur.2024.1443903 (PMC11317261; doi:10.3389/fneur.2024.1443903)
Supplement: Supplementary file 1 [file Data_Sheet_1.docx]

Supplementary Material

# S1 Table. Semiology elements of mesial temporal lobe epilepsy identification

| Elements of inclusion | Elements of exclusion |
| --- | --- |
| Abdominal aura | Auditory aura |
| Fear | The onset of earlier impairment of awareness |
| Déjà vu |  |
| Autonomic phenomena |  |
| The onset of behavioral arrest with slowly progressive impairment of awareness |  |
| Ictal speech |  |

| Variable | QOLIE-31 | Age | Sex | Employment | Seizure frequency | Number of ASMs | NDDI-E score |
| --- | --- | --- | --- | --- | --- | --- | --- |
| QOLIE-31 | 1 |  |  |  |  |  |  |
| Age | -.063 | 1 |  |  |  |  |  |
| Sex | .221 | .100 | 1 |  |  |  |  |
| Employme-nt | **.317** | .074 | -.171 | 1 |  |  |  |
| Seizure frequency | -.083 | -.030 | .007 | −.226 | 1 |  |  |
| Number of ASMs | **-.319** | .193 | -.152 | −.032 | **.435** | 1 |  |
| NDDI-E score | **-.682** | -.030 | .177 | **−.329** | -.036 | .029 | 1 |

# S2 Table. Correlation matrix in extratemporal lobe epilepsy

# Spearman rank correlation coefficient. Bold: p <0.05. QOLIE-31: Quality of Life in Epilepsy Inventory-31; ASM: antiseizure medication; NDDI-E: Neurological Disorders Depression Inventory for Epilepsy.

# S3 Table. Comparison between high and low scoring groups of the Quality of Life in Epilepsy

# Inventory-31 in extratemporal lobe epilepsy

| Characteristics | High (n = 20) | Low (n = 27) | *p* |
| --- | --- | --- | --- |
| Age (years) | 37.0 (27.0-46.0) | 37.0 (26.2-46.7) | 0.803 |
| Sex |  |  |  |
| Male | 7 (35%) | 12 (44%) | 0.561 |
| Female | 13 (65%) | 15 (56%) | 0.561 |
| Employment | 17 (85%) | 19 (70%) | 0.159 |
| Seizures frequency |  |  |  |
| None | 14 (70%) | 16 (60%) | 1.000 |
| Per year | 1 (5%) | 2 (7%) | 1.000 |
| Per month | 3 (15%) | 7 (26%) | 0.718 |
| Per day | 2 (10%) | 2 (7%) | 1.000 |
| Number of ASMs | 1.0 (1.0-3.0) | 2.0 (1.0-3.0) | 0.917 |
| NDDI-E | 9.0 (7.0-11.0) | 14.5 (11.0-16.7) | 0.031 |

High: ≥median QOLIE-31 score. Low: <median QOLIE-31 score. Median (interquartile range) or number (%). Mann–Whitney U test or chi-square test. *p* < 0.05. ASM: antiseizure medications; NDDI-E: Neurological Disorders Depression Inventory for Epilepsy.

# S4 Table. Correlation matrix in mesial temporal lobe epilepsy

| Variable | QOLIE-31 | Age | Sex | Employm-ent | Seizure frequency | Number of ASMs | NDDI-E score |
| --- | --- | --- | --- | --- | --- | --- | --- |
| Age | 1 |  |  |  |  |  |  |
| Sex | .040 | 1 |  |  |  |  |  |
| QOLIE-31 | -.008 | **-.332** | 1 |  |  |  |  |
| Employme  -nt | **.330** | **.452** | -.248 | 1 |  |  |  |
| Seizure frequency | -.295 | -.138 | .048 | **−.333** | 1 |  |  |
| Number of ASMs | **-.376** | .096 | .042 | .033 | **.351** | 1 |  |
| NDDI-E score | **-.690** | -.187 | .186 | **−.342** | **.406** | **.586** | 1 |

Spearman rank correlation coefficient. Bold: p <0.05. QOLIE-31: Quality of Life in Epilepsy Inventory-31; ASM: antiseizure medication; NDDI-E: Neurological Disorders Depression Inventory for Epilepsy.

# S5 Table. Comparison between high and low scoring groups of the Quality of Life in Epilepsy

# Inventory-31 in mesial temporal lobe epilepsy

| Characteristics | High (n = 19) | Low (n = 18) | *p* |
| --- | --- | --- | --- |
| Age (years) | 46.0 (33.0-50.0) | 41.5 (38.2-48.0) | 0.611 |
| Gender |  |  |  |
| Male | 10 (53%) | 9 (50%) | 0.517 |
| Female | 9 (47%) | 9 (50%) | 0.517 |
| Employment | 18 (95%) | 14 (78%) | 0.180 |
| Seizures frequency |  |  |  |
| None | 14 (74%) | 9 (50%) | 0.184 |
| Per year | 4 (21%) | 5 (28%) | 0.714 |
| Per month | 1 (5%) | 4 (22%) | 0.180 |
| Per day | 0 (0%) | 0 (0%) | 1.000 |
| Number of AEDs | 1.0 (1.0-1.0) | 2.0 (1.0-3.0) | 0.014 |
| NDDI-E | 8.0 (7.0-10.0) | 14.0 (11.0-17.2) | 0.002 |

High: ≥median QOLIE-31 score. Low: <median QOLIE-31 score.

Median (interquartile range) or number (%). Mann–Whitney U test or chi-square test. p < 0.05.

ASM: antiseizure medication; NDDI-E: Neurological Disorders Depression Inventory for Epilepsy.

# S6 Table. Correlation matrix for each the Quality of Life in Epilepsy Inventory-31 domains

# and antiseizure medication in extratemporal lobe epilepsy

| Variable | QOLIE-31 | Seizure worry | Emotional | Overall QOL | Energy/fatigue | Cognitive | Medical | Social | Age | Sex | ASMs |
| --- | --- | --- | --- | --- | --- | --- | --- | --- | --- | --- | --- |
| QOLIE-31 | 1 |  |  |  |  |  |  |  |  |  |  |
| Seizure worry | **.575** | 1 |  |  |  |  |  |  |  |  |  |
| Emotional | .**747** | .251 | 1 |  |  |  |  |  |  |  |  |
| Overall QOL | **.669** | **.451** | **.542** | 1 |  |  |  |  |  |  |  |
| Energy/fatigue | **.779** | **.348** | **.746** | **.535** | 1 |  |  |  |  |  |  |
| Cognitive | **.774** | **.441** | **.525** | **.307** | **.529** | 1 |  |  |  |  |  |
| Medical | **-.381** | **-.389** | -.160 | -.165 | -.151 | **-.494** | 1 |  |  |  |  |
| Social | **.838** | **.441** | **.461** | **.549** | **.579** | **.493** | **-.303** | 1 |  |  |  |
| Age | -.063 | -.179 | .108 | .110 | -.019 | -.132 | .170 | -.161 | 1 |  |  |
| Sex | .221 | .214 | .013 | .100 | .048 | .005 | -.239 | **.375** | .100 | 1 |  |
| ASMs | **-.319** | **-.292** | -.122 | -.230 | -.195 | -.125 | .270 | **-417** | .193 | -.152 | 1 |

Spearman rank correlation coefficient. Bold: *p* <0.05. QOLIE-31: Quality of Life in Epilepsy Inventory-31total score; Emotional: Emotional well-being; Cognitive: Cognitive functioning; Medical; Medical effects; Social: Social functioning; ASM: antiseizure medication; NDDI-E: Neurological Disorders Depression Inventory for Epilepsy.

# S7 Table. Comparison between high and low scoring groups on each the Quality of Life in Epilepsy Inventory-31 domains in

# extratemporal lobe epilepsy

|  | Seizure worry | | | Emotional well-being | | | | Overall QOL | | | | Energy/fatigue | | | | Cognitive functioning | | | Medical effects | | | Social functioning | | |
| --- | --- | --- | --- | --- | --- | --- | --- | --- | --- | --- | --- | --- | --- | --- | --- | --- | --- | --- | --- | --- | --- | --- | --- | --- |
|  | High (n = 28) | Low (n = 19) | *p* | High (n = 27) | Low (n = 20) | *p* | High (n = 23) | | Low (n = 24) | *p* | High (n = 28) | | Low (n = 19) | *p* | High (n = 22) | | Low (n = 25) | *p* | High (n = 27) | Low (n = 20) | *p* | High (n = 24) | Low (n = 23) | *p* |
| Age | 35.0 (27.0-44.7) | 43.0 (26.0-48.0) | 0.895 | 37.0 (27.0-46.0) | 36.5 (26.2-47.0) | 0.796 | 40.0 (26.0-46.0) | | 36.5 (28.0-46.7) | 0.473 | 35.5 (27.0-44.7) | | 43.0 (26.0-48.0) | 0.546 | 36.5 (26.7-46.2) | | 37.0 (26.5-47.0) | 0.846 | 37.0 (31.0-46.0) | 36.5 (25.2-46.7) | 0.786 | 35.5 (27.0-44.5) | 43.0 (26.0-48.0) | 0.473 |
| Sex |  |  |  |  |  |  |  | |  |  |  | |  |  |  | |  |  |  |  |  |  |  |  |
| Male | 12 (43%) | 16 (84%) | 1.000 | 12 (44%) | 15 (75%) | 1.000 | 9 (39%) | | 11 (58%) | 0.770 | 12 (43%) | | 8 (42%) | 1.000 | 9 (41%) | | 11 (44%) | 1.000 | 13 (48%) | 7 (35%) | 0.392 | 6  (25%) | 14  (61%) | 0.019 |
| Female | 16 (57%) | 11 (16%) | 1.000 | 15 (56%) | 12 (25%) | 1.000 | 14 (61%) | | 13 (42%) | 0.770 | 16 (57%) | | 11 (58%) | 1.000 | 13 (59%) | | 14 (56%) | 1.000 | 14 (52%) | 13 65%) | 0.392 | 18  (75%) | 9  (39%) | 0.019 |
| Number of ASMs | 1.0 (1.0-3.0) | 2.0 (1.0-3.0) | 0.293 | 1.0 (1.0-3.0) | 2.0 (1.0-3.0) | 0.768 | 1.0 (1.0-2.5) | | 2.0 (1.0-3.0) | 0.823 | 1.0 (1.0-3.0) | | 2.0 (1.0-3.0) | 0.917 | 1.0 (1.0-3.0) | | 2.0 (1.0-3.0) | 0.973 | 2.0 (1.0-3.0) | 1.0 (1.0-2.0) | 0.113 | 1.0 (1.0-2.0) | 2.0 (1.0-3.0) | 0.091 |

High: score ≥median score. Low: median score <median score. Median (interquartile range) or number (%). Mann–Whitney U test or chi-square test. *p* < 0.05. ASM: antiseizure medications; NDDI-E: Neurological Disorders Depression Inventory for Epilepsy.

# S8 Table. Correlation matrix for each the Quality of Life in Epilepsy Inventory-31 domains and

# antiseizure medication in mesial temporal epilepsy

| Variable | QOLIE-31 | Seizure worry | Emotional | Overall QOL | Energy/fatigue | Cognitive | Medical | Social | Age | Sex | ASMs |
| --- | --- | --- | --- | --- | --- | --- | --- | --- | --- | --- | --- |
| QOLIE-31 | 1 |  |  |  |  |  |  |  |  |  |  |
| Seizure worry | **.639** | 1 |  |  |  |  |  |  |  |  |  |
| Emotional | **.829** | **.615** | 1 |  |  |  |  |  |  |  |  |
| Overall QOL | **.769** | **.368** | **.672** | 1 |  |  |  |  |  |  |  |
| Energy/fatigue | **.770** | .317 | **.599** | **.580** | 1 |  |  |  |  |  |  |
| Cognitive | **.719** | **.398** | **.440** | **.398** | **.445** | 1 |  |  |  |  |  |
| Medical | **-.374** | -.202 | -.225 | -.202 | **-.345** | **-.363** | 1 |  |  |  |  |
| Social | **.755** | **.336** | **.574** | **.336** | **.638** | **.425** | -.270 | 1 |  |  |  |
| Age | .040 | .223 | .094 | .151 | .119 | -.025 | -.114 | -.108 | 1 |  |  |
| Sex | -.008 | -.124 | -.102 | .003 | .018 | .003 | **.332** | .056 | **-.332** | 1 |  |
| ASMs | **-.376** | **-.396** | **-.513** | -.070 | -.169 | -.297 | .061 | **-513** | .096 | .042 | 1 |

Spearman rank correlation coefficient. Bold: *p* <0.05. QOLIE-31: Quality of Life in Epilepsy Inventory-31total score; Emotional: emotional well-being; Cognitive: Cognitive functioning; Medical; Medical effects; Social: Social functioning; ASM: antiseizure medication.

# S9 Table. Comparison between high and low scoring groups of each the Quality of Life in Epilepsy Inventory-31 domains in mesial temporal lobe

# epilepsy

|  | Seizure worry | | | | Emotional well-being | | | | Overall QOL | | | | Energy/fatigue | | | | Cognitive functioning | | | | Medical effects | | | | Social functioning | | |
| --- | --- | --- | --- | --- | --- | --- | --- | --- | --- | --- | --- | --- | --- | --- | --- | --- | --- | --- | --- | --- | --- | --- | --- | --- | --- | --- | --- |
|  | High (n = 18) | Low (n = 19) | *p* | High (n = 19) | | Low (n = 18) | *p* | High (n = 23) | | Low (n = 14) | *p* | High (n = 20) | | Low (n = 17) | *p* | High (n = 20) | | Low (n = 17) | *p* | High (n = 23) | | Low (n = 14) | *p* | High  (n = 21) | | Low  (n = 16) | *p* |
| Age | 46.0 (37.5-51.5) | 41.0 (36.0-47.0) | 0.417 | 46.0 (33.0-51.0) | | 41.5 (38.2-47.2) | 0.243 | 44.0 (33.0-51.0) | | 43.0 (39.0-48.2) | 0.963 | 45.0 (36.5-49.7) | | 41.0 (35.0-48.0) | 0.602 | 43.0 (38.2-49.7) | | 44.0 (32.0-48.0) | 0.837 | 42.0 (38.0-48.0) | | 45.0 (33.7-58.5) | 0.963 | 42.0 (34.5-49.5) | | 44.5 (39.0-48.0) | 0.921 |
| Sex |  |  |  |  | |  |  |  | |  |  |  | |  |  |  | |  |  |  | |  |  |  | |  |  |
| Male | 10 (56%) | 9 (47%) | 0.746 | 10 (53%) | | 9 (50%) | 1.000 | 11 (48%) | | 8 (57%) | 0.737 | 10 (50%) | | 9 (53%) | 1.000 | 10 (50%) | | 9 (53%) | 1.000 | 7 (30%) | | 12 (86%) | 0.002 | 10 (47%) | | 9 (56%) | 0.743 |
| Female | 8 (44%) | 10 (53%) | 0.476 | 9 (47%) | | 9 (50%) | 1.000 | 12 (52%) | | 6 (43%) | 0.737 | 10 (50%) | | 8 (47%) | 1.000 | 10 (50%) | | 8 (47%) | 1.000 | 16 (70%) | | 2 (14%) | 0.002 | 11 (53%) | | 7 (44%) | 0.743 |
| Number of ASMs | 1.0 (1.0-1.2) | 2.0 (1.0-3.0) | 0.029 | 1.0 (1.0-1.0) | | 2.0 (1.0-3.0) | 0.002 | 1.0 (1.0-3.0) | | 1.0 (1.0-2.0) | 0.760 | 1.0 (1.0-2.0) | | 2.0 (1.0-2.5) | 0.298 | 1.0 (1.0-2.0) | | 2.0 (1.0-3.0) | 0.444 | 1.0 (1.0-2.0) | | 1.0 (1.0-3.0) | 0.760 | 1.0 (1.0-2.0) | | 2.0 (1.0-3.0) | 0.290 |

High: score ≥median score. Low: median score <median score. Median (interquartile range) or number (%). Mann–Whitney U test or chi-square test. *p* < 0.05. ASM: antiseizure medication.
